# Ifosfamide as a Cause of Fanconi Syndrome

**DOI:** 10.7759/cureus.22755

**Published:** 2022-03-01

**Authors:** Daniel Martinez, Joaquin Rodelo, Sebastian Pelaez García

**Affiliations:** 1 Internal Medicine, Universidad de Antioquia, Medellín, COL; 2 Nephrology, Hospital San Vicente Fundación, Medellín, COL

**Keywords:** hyperchloremic metabolic acidosis, chemotherapy, glomerulopathy, renal tubulopathy, ifosfamide, fanconi

## Abstract

Ifosfamide-induced Fanconi syndrome is a rare complication that occurs in patients in treatment with ifosfamide. It is usually characterized by type II proximal renal tubular dysfunction, as evidenced by glycosuria, proteinuria, electrolyte loss, and metabolic acidosis. We outline two case reports of patients who received ifosfamide as chemotherapy for Ewing’s sarcoma and extranodal B-cell lymphoma.

## Introduction

The reabsorption of many solutes takes place in the proximal renal tubule, with 65% of sodium, 90% of glucose, amino acids, bicarbonate, and phosphorus being absorbed there [1]. Fanconi syndrome is associated with inadequate reabsorption in the proximal renal tubule [2]. Different abnormalities lead to its suspicion: amino-aciduria (with losses up to 0.5-1 g/day), glucosuria in absence of hyperglycemia, hypophosphatemia due to reabsorption disruption, natriuresis, kaliuresis, and hyperchloremic metabolic acidosis [1-3].

The most common cause of Fanconi syndrome in adults is exposure to endogenous or exogenous toxins such as heavy metals, drugs, or dysproteinemias. Inborn errors of metabolism are the most common cause in children [1]. Many drugs have been associated with Fanconi syndrome, such as antibiotics, antiviral therapy drugs, anticonvulsants, and chemotherapy drugs such as ifosfamide (which is used for hematological and non-hematological malignancies) [4]. Ifosfamide-related Fanconi syndrome has been described in up to 5-7% of patients treated with this drug [5]. It may also cause nephrotoxicity, proximal tubulopathy, and glomerulopathy [6].

We outline two case reports of patients who received ifosfamide as chemotherapy, who presented with metabolic abnormalities compatible with Fanconi syndrome.

## Case presentation

Case #1

A 23-year-old male patient, residing in an urban area, was diagnosed with Ewing’s sarcoma in 2018 with staging T2N1Mx. The patient underwent chemotherapy with vincristine, doxorubicin, cyclophosphamide intercalated with ifosfamide and etoposide every 2-3 weeks (Table 1); after nine cycles doxorubicin was exchanged for actinomycin, and the patient received three more cycles of chemotherapy. 

**Table 1:** Chemotherapy cycle

Chemotherapy cycle	Days
Vincristine 2mg + Doxorubicin 123mg + Cyclophosphamide 2000mg (Doxorubicin was then exchanged for actinomycin 2mg) (First part)	Day 1
Ifosfamide 2916mg + etoposide 162mg (Second part, 2-3 weeks after first part)	Day 1 to 5

**Figure 1:**
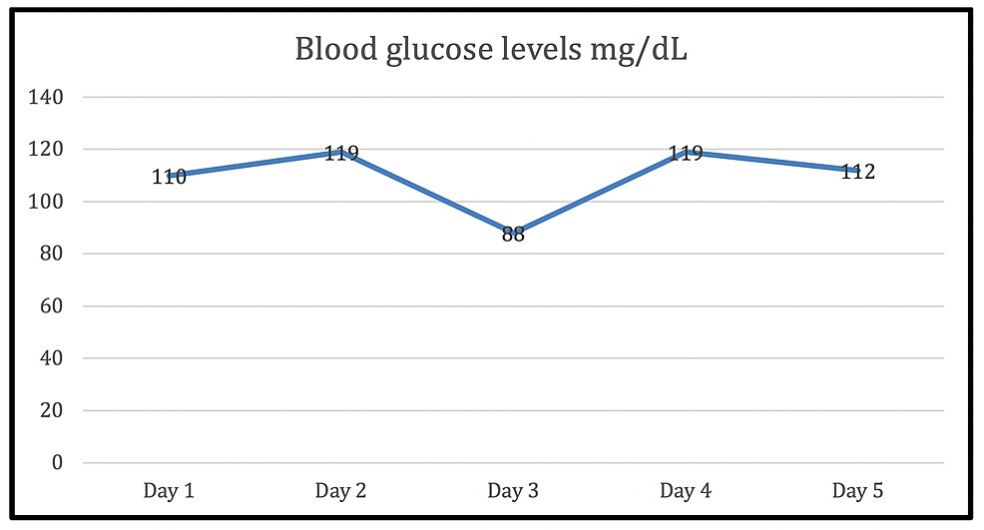
Blood glucose levels

He showed tumor progression on the upper right limb which required disarticulation surgery and the restart of chemotherapy for six additional cycles. The last cycle was performed a week after an outpatient follow-up at Hospital San Vicente Fundación, in Medellín. Follow-up echocardiography showed two moving, hypoechogenic masses attached to the postero-medial papillary muscle, of sizes 8.6x4.5mm and 7.2x5.3mm. Therefore, the patient was admitted to rule out endocarditis. Chest and abdomen computed tomography (CT) and bone scintigraphy were performed, with no metastatic involvement; aerobic and anaerobic blood cultures were negative. Further workup showed creatinine levels to be at 1.14mg/dL. The glomerular filtration rate (eGFR) was estimated through the CKD-EPI equation (Chronic Kidney Disease Epidemiology Collaboration) to be 90.14 ml/min/1,73 m²); his urinalysis showed 1000mg/dL glucosuria and 75mg/dL proteinuria, with blood glucose less than 180mg/dl (Figure 1) and glycated hemoglobin A1c of 5.9%, which is in the pre-diabetes range. A tubulopathy was suspected, hence, arterial blood gas analysis was performed, which showed the pH to be 7.3, HCO3 to be 23.1 mmol/L, PCO2 to be 47.7 mmHg, and anion gap to be 12 (respiratory acidosis + metabolic acidosis with normal anion gap). Serum uric acid was found to be low at 2.1 mg/dL; the 24-hour urine protein was found to be at 1126 mg, with fractional excretion of phosphate to be at 30.7% and fractional excretion of potassium to be at 11.1%. Tubulopathy was ruled out and Fanconi syndrome diagnosis was inferred due to Ifosfamide use. The oncology department withdrew the offending drug and nephrology prescribed oral bicarbonate therapy for two months. Later the arterial blood gases were found to be normal, showing a pH 7.43, PCO2 of 30 mmHg, HCO3 of 22.3 mmol/L, and normal levels of potassium. Months later, cardiac masses were surgically resected, with the pathology department discovering metastatic involvement; new imaging studies demonstrated liver, pancreatic, left lung, gallbladder wall, bilateral paravertebral, and gluteus muscle involvement. Consequently, the patient underwent palliative care. The initial respiratory acidosis was not investigated but it could have been secondary to consumption of opioid analgesics or decreased muscle mass.

Case #2

A 44-year-old female patient, residing in an urban area, was diagnosed with aggressive extranodal B-cell lymphoma, with metastasis to the spine, pelvic ring, right femur, and pathologic L4 fracture and secondary nerve compression, in January 2019. She initially received six R-CHOP cycles (Rituximab, cyclophosphamide, doxorubicin, vincristine, and prednisone), 10 lumbar and three cervical radiotherapy sessions, which were discontinued due to a right hip fracture in December 2019, which required a total prosthetic replacement. Later, a pathology report showed relapse (germinal center diffuse large cell lymphoma). 

A positron emission tomography (PET) performed in January 2020 showed countless lytic lesions on axial and appendicular bones. Hence, second-line therapy was started (rituximab, ifosfamide, carboplatin, and etoposide). Three days after second-line therapy was started, the patient presented with a febrile syndrome with no focus, therefore cefepime and vancomycin were started. However, no microbiological isolation was made and laboratory results showed low serum uric acid at 1.4 mg/dL, low serum phosphate at 1.8 mg/dL, normal lactic acid at 3.3 mg/dL, low magnesium at 1.3 mg/dL. Arterial blood gas analysis showed pH 7.28, HCO3 12.1 mmol/L, PCO2 18 mmHg, sodium 142 mmol/L, chloride 119 mmol/L, and potassium at 3.1 mmol/L. Metabolic acidosis with normal anion gap was diagnosed, the renal function was preserved (creatinine 0.64 mg/dL, with GFR through 2021 CKD-EPI of 108 mL/min/1,73 m2); urinalysis showed proteinuria (Table 2). Urinary electrolytes revealed urinary anion gap: sodium 128 mmol/L, potassium 11.2 mmol/L, chloride 111 mmol/L. Urinary anion gap was positive at 28, however, urinary pH was > 6.5, which could have altered urinary anion gap results.

**Table 2:** Urinalysis and urinary sediment

Characteristic	Value	Reference range
Aspect	Clear	Clear/normal
Color	Yellow	
Glucose	Normal	
Proteins (mg/dL)	25	Negative
Bilirrubin (mg/dL)	Normal	Normal
Urobilinogen (mg/dL)	Normal	Normal
pH	7	5-6
Density	1010	1010 – 1020
Blood (RBC/uL)	10	Negative
Ketonic bodies (mg/dL)	5	Negative
Nitrites	Negative	Negative
Leukocyte esterase	Negative	Negatve
Urinary Sediment		
Red blood cells	None observed	
Leukocytes	2/HPF	
Bacteria	None observed	
Epithelial cells	1/HPF	
Mucus	Scarce amount	

Nephrology concluded that the metabolic acidosis with a normal anion gap, hypokalemia, low uric acid, hypophosphatemia, hypomagnesemia, and proteinuria with no GFR disturbance was compatible with Fanconi syndrome, probably related to Ifosfamide use as previous laboratory values were normal. Intravenous bicarbonate therapy was started for one week, with acid-base and electrolyte normalization. And the Fanconi syndrome was found to be reversible. Oncology withdrew chemotherapy and third-line therapy was started with R-DHAOx (Rituximab, dexamethasone, cytarabine, oxaliplatin) for outpatient management. 

## Discussion

Fanconi syndrome is a disorder of the proximal tubule, which leads to malabsorption of electrolytes and other substances [1]. It may be acquired or inherited. It’s usually acquired in adults. Its hereditary causes are cystinosis, galactosemia, mitochondrial diseases, fructose intolerance, tyrosinemia, Wilson’s disease, and idiopathic ones. Among purchased drugs (Table 3), monoclonal gammopathies or toxic induced ones [7]. 

**Table 3:** Drugs Adapted from [8]

Drugs associated with Fanconi syndrome	
Nucleoside reverse transcriptase inhibitors	Lamivudine, stavudine
Acyclic nucleoside phosphonates	Cidofovir, adefovir, tenofovir
Aminoglycosides	Gentamicin, tobramycin, amikacin
Alkylating agents and platinum agents	Ifosfamide, cisplatin, carboplatin
Anticonvulsants	Valproic acid
Antiparasitics	Suramin
Histamine 2 blockers	Ranitidine, cimetidine
Carbonic anhydrase inhibitors	Acetazolamide
Salicylate intoxication	Acetylsalicylic acid
Iron chelators	Deferasirox

Herein, we described two cases of ifosfamide-associated Fanconi syndrome. Among chemotherapy agents, ifosfamide is classified as an alkylating agent. Its active metabolites alkylate or bind with intracellular structures, including nucleic acids. Its cytotoxic action is mainly due to its binding to DNA and RNA, with protein synthesis inhibition. Ifosfamide is used as a chemotherapy agent for the treatment of sarcomas, lymphomas, breast cancer, testicular or pulmonary cancer. However, its use may have certain complications such as proximal tubulopathy, and Fanconi syndrome (whether it is associated with diabetes insipidus or not). The first case had glucosuria with normal serum glucose, metabolic acidosis with a normal anion gap, low serum uric acid, proteinuria, and increased phosphate and potassium excretion (Table 4). Hence, Fanconi syndrome was diagnosed. Even though it was an unlikely cause due to the age and lack of personal history of heavy metal intoxication; nor were tetracycline, antiviral, or azathioprine use was recorded; no monoclonal gammopathy findings were observed. The second patient presented with metabolic acidosis with a normal anion gap, hypokalemia, low uric acid, hypophosphatemia, hypomagnesemia, and proteinuria, with no renal function impairment (Table 4). Remarkably, a urinary anion gap was positive, although urinary pH was > 6.5, which could lead to an unreliable urinary anion gap; however, it may also be associated with a concomitant type 1 tubular acidosis. Hereditary causes were unlikely, as admission laboratory workup was normal, no evidence of monoclonal gammopathy was observed, and the whole clinical picture started after chemotherapy and two antibiotic exposures (cefepime and vancomycin); the last two agents have not been associated with Fanconi syndrome or type 1 tubular acidosis. Among chemotherapy agents, first-line drugs were unlikely to be the cause as no laboratory abnormalities were observed. The second-line drugs were likely to be associated they were administered before the clinical presentation. Among second-line agents, Ifosfamide is strongly associated with both Fanconi syndrome and type 1 tubular acidosis. Therefore, it was considered as the most likely cause. The drug was withdrawn, electrolytes were replenished, and intravenous bicarbonate therapy was started, with normalization of laboratory values. 

**Table 4:** Laboratory values.

Laboratory values for both patients		
Test	Case #1	Case #2
Creatinine mg/dL	1.14	0.64
Glucosuria mg/dL	1000	Negative
Proteinuria orina ocasional mg/dL	75	25
pH	7.3	7.28
HCO3 mmol/L	23.1	12.1
PCO2 mmHg	47.7	18
Anion gap	12	11
Serum uric acid mg/dL	2.1	1.4
Phosphate mg/dL		1.8

The mechanism underlying ifosfamide-induced Fanconi syndrome isn’t well established. As it is a pro-drug and it needs to be activated by the hepatic cytochrome oxidase. A theory related to ifosfamide metabolism-related products has been proposed [9]. These products are selectively taken by the organic cation transporter 2 (OCT2), which is found on the basal-lateral aspect of the proximal tubule cells; a reduction of dehydrogenase has also been observed [10]. Additionally, chloroacetaldehyde metabolite accumulates on the proximal tubule cells and reduces antioxidant glutathione and adenosine triphosphate levels are involved. This process is related to endocytosis and intracellular protein processing reduction [11]. 

In conclusion, ifosfamide, just as cyclophosphamide, is an effective drug against malignant neoplasms; however, nephrotoxic effects are common, and these may be permanent and irreversible. Strict follow-up of renal and tubular function is mandatory (urinalysis, serum, and urine electrolyte measurement, arterial blood gas analysis).

## Conclusions

The most common causes of Fanconi syndrome in adults are: endogenous or exogenous toxins such as heavy metals, drugs, or dysproteinemias. Among drugs, plenty have been associated with Fanconi syndrome, such as: antibiotics, antiviral therapy, anticonvulsants, and chemotherapy such as ifosfamide. It may also cause nephrotoxicity, proximal tubulopathy and glomerulopathy. Patients who receive ifosfamide must be closely monitored for renal impairment to avoid this rare but fatal complication. 
